# Associations Between Body Composition, Leptin, and Vitamin D Varied by the Body Fat Percentage in Adolescents

**DOI:** 10.3389/fendo.2022.876231

**Published:** 2022-06-03

**Authors:** Rapheeporn Khwanchuea, Chuchard Punsawad

**Affiliations:** School of Medicine, Walailak University, Nakhonsithammarat, Thailand

**Keywords:** body fat percentage (BF%), leptin, 25-hydroxyvitamin D, adolescents, body composition

## Abstract

**Background:**

Serum leptin levels reflects one’s degree of obesity and can affect vitamin D levels. The relationship between body fat, leptin, and 25-hydroxyvitamin D (25(OH)D) has not been extensively studied in adolescents. This study aimed to investigate the correlations between body composition and leptin and 25(OH)D levels in boys and girls.

**Methods:**

Participants aged 12–14 years (n = 205) were grouped according to sex. After body composition was recorded using bioelectrical impedance analysis, they were classified into three groups according to body fat percentage (%BF) (< 30, ≥ 30 and < 40, and ≥ 40). Serum leptin and 25(OH)D levels were measured using the enzyme-linked immunosorbent assay (ELISA). Correlations between all variables were analyzed according to sex and the percentage of BF groups.

**Results:**

Boys and girls with %BF ≥ 30 showed no difference in body mass index (BMI), %BF, and leptin and 25(OH)D, while other variables of body composition were more common in boys than in girls. The %BF, body fat mass (BFM), and 25(OH)D of both sexes with %BF ≥ 30, and leptin levels of boys with %BF ≥ 40 increased with an increase in %BF. A negative correlation between leptin and 25(OH)D levels was found in boys with %BF < 40 and girls with %BF < 30. In the %BF ≥ 30 and < 40 groups, there were negative correlations between leptin, BFM, free fat mass, and muscle mass (MM); between leptin, 25(OH)D, and height in boys; and between 25(OH)D, body weight, BMI, and MM in girls.

**Conclusion:**

A negative correlation between leptin and 25(OH)D levels varied according to sex, while for body composition, it was evident at 30 and 40% BF.

## Introduction

Obesity is defined as an excessive proportion of body fat relative to lean body mass due to a chronic imbalance between energy intake and expenditure. Leptin resistance, or its inability to modulate energy intake and expenditure is common in obesity. Leptin is an adipokine secreted primarily by adipocytes, and it inhibits energy intake and regulates energy homeostasis by acting *via* hypothalamic receptors (1, 2). Research shows that leptin is a marker of obesity and reflects the degree of adiposity. Circulating leptin concentrations are determined by body fat mass (BFM), body mass index (BMI), and sex. In fact, leptin concentration is higher in females than in males regardless of BFM (1, 3, 4). In addition, leptin levels change significantly during progressive pubertal stages, with girls having higher serum leptin levels than boys which rise throughout puberty, concomitant with an increase in estrogen levels (4). Furthermore, serum leptin concentrations are higher in early adolescence than in childhood and may play a role in pubertal development (4). Body fat percentage (%BF), basal metabolic rate, muscle mass (MM), bone mass, and serum 25-hydroxyvitamin D (25(OH)D) had an impact on serum leptin (5).

In obesity, there is not only an imbalance of adipokines, but also a decrease in vitamin D bioavailability (6, 7). Adipose tissue is a target for vitamin D and the main storage depot for vitamin D and its metabolites (8, 9). Leptin exerts an autocrine–paracrine lipolytic effect on adipocytes by interacting with the vitamin D receptor, and it inhibits the enzyme that converts 25(OH)D to 1,25 dihydroxyvitamin D (10). Thus, vitamin D depletion might increase appetite and lead to obesity by directly regulating leptin expression (6, 11). Previous studies reported that there was a negative association between %BF and vitamin D and a positive correlation between %BF and leptin that confirmed excess of %BF, leading to decreased vitamin D and raised leptin (12). The distribution of fat in adolescents with BMI of 36 ± 5 and %BF of 40 ± 5 might be associated with vitamin D status with decreased 25(OH)D (13). A recent study in young adults aged 20–21 years found that males and females demonstrated positive relationships between serum leptin and BMI, waist circumference, and %BF; however, males showed inverse correlations between serum leptin, MM, and 25(OH)D (5). In adults, the relationship between total body fat and 25(OH)D levels also varied by sex (14). In addition, the relationship between serum 25(OH)D and leptin is largely explained by the presence of adiposity or the amount of body fat, which disappeared after adjustment for total body fat and waist circumference (15).

Although the inverse relationship between leptin and 25(OH)D has been reported with respect to BMI and %BF, this association has not been fully explored in young adolescents. Moreover, there was a report in Thai school children aged 6–14 years that dietary calcium intake was low, vitamin D status was sufficient, and girls experienced a decline in 25(OH)D levels with increasing age (16). Thus, this study investigated the correlations between body composition and leptin and 25(OH)D levels in boys and girls stratified degree of obesity by %BF.

## Materials and Methods

### Study Participants

Students with ages 12–14 years and BMI-for-age > 50^th^ percentile (17) were selected from high schools in southern Thailand. Those with chronic diseases, other conditions such as asthma, allergies, or gastritis, and those using steroids were excluded. Participants and their parents received the information regarding the purposes and methods of the study, and they were required to sign the informed consent. Participants (n = 205) were grouped by sexes (107 boys and 98 girls), and after body composition measurement, they were classified by %BF into three groups, group 1, %BF < 30 (28 boys and 12 girls); group 2, %BF ≥ 30 and < 40 (38 boys and 43 girls); group 3, %BF ≥ 40 (41 boys and 43 girls), according to the %BF cutoff values at 95^th^ percentile for age, 30% (18, 19) and 40% (20). Participants were asked about their lifestyle habits, and they did not exercise regularly; during the day they spent most of their time sitting in class and ate three meals a day with snacks and no calcium and vitamin D supplementation (21).

This study was reviewed and approved by the Human Research Ethics Committee of Walailak University, Thailand (Approval Number: WUEC-19-102-21). A consent form was obtained from all participants or their legal representative before enrollment.

### Anthropometric and Body Composition Measurements

Body composition, including body weight (BW, kg), BMI (kg/m^2^), %BF, BFM (kg), free fat mass (FFM, kg), and MM (kg), were analyzed by bioelectrical impedance analysis (22, 23) using a TANITA SC-330ST series body composition analyzer (Tanita Corporation, Tokyo, Japan). To increase measurement accuracy, participants wore light clothes and no shoes. Since the level of hydration, the presence of edema, and the daily weight variability affected the total body weight, 0.5 kg was subtracted from the obtained weight values (23). Standing heights (m) were measured without shoes using a locally made stadiometer and were recorded to the nearest 0.1 cm (21).

### Measurements of Leptin and 25-Hydroxyvitamin D by Enzyme-Linked Immunosorbent Assay (ELISA)

After an overnight fast, participants were collected venous blood samples in clotted blood tubes. The blood samples were centrifuged at 2000 revolutions per minute for 10 minutes, and the serum was harvested into 1.5 mL microcentrifuge tubes and stored at -70°C until used to measure leptin and 25(OH)D.

Leptin and 25(OH)D levels were determined by an immunometric sandwich ELISA using commercial ELISA kits leptin (R&D Systems, Inc., Minneapolis, MN, USA, sensitivity to 7.8 pg/mL and intra-assay coefficient of variability < 10%), and 25(OH)D (DRG Diagnostics, Frauenbergstrasse, Germany, sensitivity to 2.89 ng/mL and intra-assay coefficient of variability < 10%), per the manufacturer’s instructions. Standard or diluted serum samples were prepared and incubated in coated microplates at room temperature. Immunoassays were performed in duplicates. After washing, a mixture of capture and detector antibodies was added to the plate and incubated at room temperature. A 3,3′,5,5′-tetramethylbenzidine (TMB) substrate was added to each well and incubated for 10 minutes to detect the antigen-antibody complex reaction. Finally, a stop solution was added to each well, and the optical density (OD) was measured at 450 nm using a microplate reader.

### Statistical Analysis

Descriptive data of all variables were presented as mean ± standard deviation (S.D). Differences in variables of body composition, leptin, and 25(OH)D among sexes and %BF groups were compared by independent-samples T-test. The correlations between those variables were calculated by Pearson’s correlation coefficient (*r*). The *P* values less than 0.05, 0.01, or 0.001 were considered statistically significant. Data analysis was performed using IBM SPSS statistics version 22.0 software license authorization wizard.

## Results

**Table 1** shows that for all participants, BMI, %BF, BFM, and 25(OH)D levels were not different between boys and girls, whereas boys had BW, height, FFM, and MM greater than girls, and girls had higher leptin levels than boys. When participants were divided into three groups, similar results were obtained for all groups: the BW, height, and MM of boys were higher than those of girls. In group 1, girls had higher %BF and leptin levels, and lower FFM than boys. In group 2, boys had more BFM and FFM than girls. In group 3, the BFM of boys was greater than that of girls. Although, serum 25(OH)D levels were not different between boys and girls in all groups, they appeared to be slightly less than 20 ng/mL in groups 1 and 2 of girls. Furthermore, **Table 2** shows that BW, BMI, %BF, and BFM of both sexes increased with increasing %BF, but their heights did not differ. Boys and girls in group 3 had higher 25(OH)D levels than those in group 1 and 2. The FFM and MM of group 1 were greater than those of groups 2 and 3 in girls, while leptin levels in group 3 were higher than those in group 1 in boys.

**Table 1:** Characteristics of study participants and comparisons^1^ of the variables between boy and girl.

Variable^2^	Sex	Total participants(107 boys and 98 girls)		Group 1 (%BF < 30)(28 boys and 12 girls)		Group 2 (%BF ≥ 30 and < 40)(38 boys and 43 girls)		Group 3 (%BF ≥ 40)(41 boys and 43 girls)	
		Mean ± S.D.	*P* value	Mean ± S.D.	*P* value	Mean ± S.D.	*P* value	Mean ± S.D.	*P* value
Age (year)	Boy	13.36 ± 0.61	0.427	13.16 ± 0.69	0.983	13.39 ± 0.55	0.318	13.39 ± 0.63	0.760
	Girl	13.29 ± 0.65		13.17 ± 0.84		13.26 ± 0.63		13.35 ± 0.61	
BW (kg)	Boy	75.18 ± 14.74	0.002	60.35 ± 6.55	< 0.001	72.97 ± 10.66	0.003	87.36 ± 11.45	< 0.001
	Girl	69.35 ± 12.44		51.43 ± 3.84		66.43 ± 8.67		77.27 ± 10.66	
Height (m)	Boy	1.66 ± 0.07	< 0.001	1.66 ± 0.06	< 0.001	1.64 ± 0.07	0.002	1.67 ± 0.07	< 0.001
	Girl	1.59 ± 0.06		1.58 ± 0.06		1.60 ± 0.06		1.59 ± 0.06	
BMI (kg/m^2^)	Boy	27.36 ± 4.52	0.850	21.86 ± 1.87	0.066	26.84 ± 1.82	0.103	31.60 ± 2.97	0.050
	Girl	27.25 ± 4.14		20.74 ± 1.29		26.00 ± 2.64		30.31 ± 2.97	
%BF	Boy	36.79 ± 11.91	0.200	21.77 ± 4.42	0.037	35.89 ± 2.32	0.597	47.88 ± 8.55	0.080
	Girl	38.58 ± 7.71		25.39 ± 5.79		35.59 ± 2.69		45.25 ± 4.12	
BFM (g)	Boy	29.12 ± 14.18	0.325	13.29 ± 3.74	0.934	26.27 ± 4.82	0.014	42.58 ± 11.54	0.001
	Girl	27.47 ± 9.52		13.18 ± 3.50		23.74 ± 4.22		35.19 ± 7.41	
FFM (g)	Boy	45.94 ± 6.87	< 0.001	46.95 ± 4.30	< 0.001	45.62 ± 7.53	0.041	45.55 ± 7.69	0.607
	Girl	41.88 ± 4.86		38.24 ± 2.32		42.70 ± 5.01		42.07 ± 4.85	
MM (g)	Boy	43.83 ± 6.01	< 0.001	44.46 ± 4.05	< 0.001	44.15 ± 5.84	0.001	43.11 ± 7.22	0.006
	Girl	39.26 ± 4.46		35.93 ± 2.14		40.02 ± 4.60		39.44 ± 4.45	
Leptin (ng/mL)	Boy	22.32 ± 5.18	0.049	19.95 ± 6.89	0.007	22.59 ± 4.21	0.380	23.70 ± 4.07	0.643
	Girl	23.59 ± 3.97		25.08 ± 4.17		23.45 ± 4.48		23.32 ± 3.32	
25(OH)D (ng/mL)	Boy	22.24 ± 5.69	0.102	21.15 ± 5.70	0.396	21.19 ± 7.06	0.248	23.97 ± 3.57	0.174
	Girl	21.06 ± 4.49		19.49 ± 5.26		19.62 ± 4.68		22.94 ± 3.31	

^1^Independent-samples T-test was used to compare the differences in mean ± S.D. of variables between sexes.

^2^S.D., Standard deviation; BW, body weight; BMI, body mass index; %BF, body fat percentage; BFM, body fat mass; FFM, fat free mass; MM, muscle mass; 25(OH)D, 25-hydroxyvitamin D.

**Table 2:** Comparisons^1^ of the variables between %BF groups^2^ of boys and girls.

Variable^3^	*P* value					
	Boys (n = 107)			Girls (n = 98)		
	Group 1 and 2	Group 1 and 3	Group 2 and 3	Group 1 and 2	Group 1 and 3	Group 2 and 3
BW (kg)	< 0.001	< 0.001	< 0.001	< 0.001	< 0.001	< 0.001
Height (m)	0.326	0.548	0.096	0.267	0.330	0.898
BMI (kg/m^2^)	< 0.001	< 0.001	< 0.001	< 0.001	< 0.001	< 0.001
%BF	< 0.001	< 0.001	< 0.001	< 0.001	< 0.001	< 0.001
BFM (g)	< 0.001	< 0.001	< 0.001	< 0.001	< 0.001	< 0.001
FFM (g)	0.370	0.337	0.964	< 0.001	< 0.001	0.558
MM (g)	0.808	0.374	0.488	< 0.001	< 0.001	0.555
Leptin (ng/mL)	0.080	0.013	0.239	0.262	0.131	0.883
25(OH)D (ng/mL)	0.979	0.025	0.033	0.938	0.008	< 0.001

^1^Independent-samples T-test was used to compare the differences in mean ± S.D. of variables (**Table 1**) between %BF groups.

^2^Group 1, %BF < 30; Group 2, %BF ≥ 30 and < 40; Group 3, %BF ≥ 40.

^3^BW, body weight; BMI, body mass index; %BF, body fat percentage; BFM, body fat mass; FFM, fat free mass; MM, muscle mass; 25(OH)D, 25-hydroxyvitamin D.

**Table 3** shows that in the group made up of only boys, BW and height were positively correlated with 25(OH)D as well as all variables of body composition (except between height and %BF), and that BMI was positively correlated with %BF (*r* = 0.938, *p* < 0.001), BFM (*r* = 0.963, *p* < 0.001), leptin (*r* = 0.195, *p* = 0.044), and 25(OH)D (*r* = 0.252, *p* = 0.009). Leptin and 25(OH)D levels positively correlated with %BF (*r* = 0.212, *p* = 0.029 and *r* = 0.212, *p* = 0.028, respectively), leptin levels negatively correlated with 25(OH)D levels (*r* = -0.35, *p* < 0.001), and 25(OH)D levels positively correlated with BFM (*r* = 0.262, *p* = 0.006) and FFM (*r* = 0.193, *p* = 0.046). In group 1, BW and BMI positively correlated with all variables of body composition. Height positively correlated with FFM (*r* = 0.885, *p* < 0.001) and MM (*r* = 0.885, *p* < 0.001), while leptin negatively correlated with 25(OH)D (*r* = -0.64, *p* < 0.001). In group 2, BW, height, and BMI positively correlated with all body composition variables; BW and height negatively correlated with leptin (*r* = -0.34, *p* = 0.037 and *r* = -0.388, *p* = 0.016, respectively); while height positively correlated with 25(OH)D (*r* = 0.334, *p* = 0.04). Leptin levels in group 2 negatively correlated with all variables of body composition (except %BF), and 25(OH)D (*r* = -0.5, *p* = 0.001). Additionally, 25(OH)D levels and BFM were positively correlated (*r* = 0.334, *p* = 0.04). In group 3, there were positive correlations between BW and %BF (*r* = 0.45, *p* = 0.003), BFM (*r* = 0.731, *p* < 0.001), as well as height with FFM and MM (*r* = 0.719, *p* < 0.001), while BMI was positively correlated with %BF (*r* = 0.799, *p* < 0.001) and BFM (*r* = 0.899, *p* < 0.001), but negatively correlated with FFM (*r* = -0.328, *p* = 0.036) and MM (*r* = -0.326, *p* = 0.037). However, there were no correlations between body composition, and leptin and 25(OH)D levels in group 3.

**Table 3:** Correlation coefficient, *r* (*P* value), between BMI parameters, body composition, leptin, and 25(OH)D in boys.

Variable^1^	%BF	BFM (g)	FFM (g)	MM (g)	Leptin (ng/mL)	25(OH)D (ng/mL)
	*r* (*P* value)	*r* (*P* value)	*r* (*P* value)	*r* (*P* value)	*r* (*P* value)	*r* (*P* value)
Total Boys (n = 107)						
BW (kg)	0.779 (< 0.001)	0.891 (< 0.001)	0.271 (0.005)	0.289 (0.003)	0.112 (0.249)	0.327 (0.001)
Height (m)	0.001 (0.992)	0.220 (0.023)	0.735 (< 0.001)	0.791 (< 0.001)	-0.090 (0.359)	0.296 (0.002)
BMI (kg/m^2^)	0.938 (< 0.001)	0.963 (< 0.001)	-0.027 (0.780)	-0.032 (0.745)	0.195 (0.044)	0.252 (0.009)
Leptin (ng/mL)	0.212 (0.029)	0.179 (0.065)	-0.115 (0.237)	-0.092 (0.347)	1.000	-0.350 (< 0.001)
25(OH)D (ng/mL)	0.212 (0.028)	0.262 (0.006)	0.193 (0.046)	0.187 (0.053)	-0.350 (< 0.001)	1.000
Group 1: Boys with %BF < 30 (n = 28)						
BW (kg)	0.614 (0.001)	0.815 (< 0.001)	0.851 (< 0.001)	0.852 (< 0.001)	-0.024 (0.905)	0.263 (0.176)
Height (m)	-0.102 (0.607)	0.157 (0.424)	0.885 (< 0.001)	0.885 (< 0.001)	-0.073 (0.713)	0.257 (0.186)
BMI (kg/m^2^)	0.892 (< 0.001)	0.938 (< 0.001)	0.377 (0.048)	0.378 (0.047)	0.064 (0.747)	0.072 (0.717)
Leptin (ng/mL)	-0.052 (0.793)	-0.027 (0.890)	0.017 (0.930)	0.014 (0.943)	1.000	-0.640 (< 0.001)
25(OH)D (ng/mL)	0.142 (0.470)	0.191 (0.330)	0.220 (0.261)	0.224 (0.252)	-0.640 (< 0.001)	1.000
Group 2: Boys with %BF ≥ 30 and < 40 (n = 38)						
BW (kg)	0.439 (0.006)	0.951 (< 0.001)	0.777 (< 0.001)	0.971 (< 0.001)	-0.340 (0.037)	0.311 (0.057)
Height (m)	0.322 (0.049)	0.871 (< 0.001)	0.755 (< 0.001)	0.952 (< 0.001)	-0.388 (0.016)	0.334 (0.040)
BMI (kg/m^2^)	0.540 (< 0.001)	0.920 (< 0.001)	0.680 (< 0.001)	0.860 (< 0.001)	-0.220 (0.184)	0.243 (0.142)
Leptin (ng/mL)	-0.146 (0.382)	-0.326 (0.046)	-0.357 (0.028)	-0.322 (0.049)	1.000	-0.500 (0.001)
25(OH)D (ng/mL)	0.227 (0.170)	0.334 (0.040)	0.243 (0.141)	0.271 (0.100)	-0.500 (0.001)	1.000
Group 3: Boys with %BF ≥ 40 (n = 41)						
BW (kg)	0.450 (0.003)	0.731 (< 0.001)	0.153 (0.338)	0.154 (0.335)	-0.079 (0.625)	0.222 (0.163)
Height (m)	-0.260 (0.100)	0.108 (0.500)	0.719 (< 0.001)	0.719 (< 0.001)	0.121 (0.452)	0.224 (0.160)
BMI (kg/m^2^)	0.799 (< 0.001)	0.899 (< 0.001)	-0.328 (0.036)	-0.326 (0.037)	-0.155 (0.334)	0.138 (0.389)
Leptin (ng/mL)	-0.097 (0.544)	-0.067 (0.675)	0.050 (0.755)	0.050 (0.757)	1.000	-0.043 (0.792)
25(OH)D (ng/mL)	0.005 (0.973)	0.093 (0.563)	0.204 (0.202)	0.203 (0.203)	-0.043 (0.792)	1.000

^1^BW, body weight; BMI, body mass index; %BF, body fat percentage; BFM, body fat mass; FFM, fat free mass; MM, muscle mass; 25(OH)D, 25-hydroxyvitamin D.

**Table 4** shows that in a group of total girls, BW, height, and BMI were positively correlated with all variables of body composition, while %BF and BFM were positively correlated with 25(OH)D levels (*r* = 0.302, *p* = 0.002 and *r* = 0.279, *p* = 0.005). In group 1, there were positive correlations of BW and height with %BF (*r* = 0.630, *p* = 0.028 and *r* = 0.591, *p* = 0.043) and BFM (*r* = 0.804, *p* = 0.002 and *r* = 0.661, *p* = 0.019), and a negative correlation between leptin and 25(OH)D (*r* = -0.784, *p* = 0.003). In group 2, BW and BMI were positively correlated with all variables of body composition and negatively correlated with 25(OH)D (*r* = -0.329, *p* = 0.031 and *r* = -0.373, *p* = 0.014), while height positively correlated with BFM (*r* = 0.511, *p* < 0.001), FFM (*r* = 0.711, *p* < 0.001) and MM (*r* = 0.711, *p* < 0.001). FFM and MM were negatively correlated with 25(OH)D (*r* = -0.353, *p* = 0.02). In group 3, there were strong positive correlations between BW, height, BMI, and all variables of body composition except between height and %BF, but no correlations between body composition variables, leptin, and 25(OH)D.

**Table 4:** Correlation coefficient, *r* (*P* value), between BMI parameters, body composition, leptin, and 25(OH)D in girls.

Variable^1^	%BF	BFM (g)	FFM (g)	MM (g)	Leptin (ng/mL)	25(OH)D (ng/mL)
	*r* (*P* value)	*r* (*P* value)	*r* (*P* value)	*r* (*P* value)	*r* (*P* value)	*r* (*P* value)
Total Girls (n = 98)						
BW (kg)	0.763 (< 0.001)	0.937 (< 0.001)	0.723 (< 0.001)	0.723 (< 0.001)	-0.004 (0.969)	0.183 (0.072)
Height (m)	0.139 (< 0.001)	0.350 (< 0.001)	0.713 (< 0.001)	0.713 (< 0.001)	0.079 (0.437)	0.060 (0.558)
BMI (kg/m^2^)	0.843 (< 0.001)	0.935 (< 0.001)	0.501 (< 0.001)	0.500 (< 0.001)	-0.048 (0.639)	0.179 (0.078)
Leptin (ng/mL)	-0.091 (0.375)	-0.026 (0.798)	0.038 (0.710)	0.039 (0.702)	1.000	-0.193 (0.057)
25(OH)D (ng/mL)	0.302 (0.002)	0.279 (0.005)	-0.076 (0.457)	-0.077 (0.453)	-0.193 (0.057)	1.000
Group 1: Girls with %BF < 30 (n = 12)						
BW (kg)	0.630 (0.028)	0.804 (0.002)	0.442 (0.150)	0.437 (0.155)	0.218 (0.496)	-0.205 (0.524)
Height (m)	0.591 (0.043)	0.661 (0.019)	0.093 (0.775)	0.092 (0.777)	0.050 (0.877)	-0.017 (0.959)
BMI (kg/m^2^)	0.015 (0.964)	0.136 (0.674)	0.415 (0.180)	0.411 (0.185)	0.193 (0.547)	-0.206 (0.520)
Leptin (ng/mL)	-0.191 (0.552)	-0.078 (0.810)	0.478 (0.116)	0.484 (0.110)	1.000	-0.784 (0.003)
25(OH)D (ng/mL)	0.056 (0.863)	-0.024 (0.940)	-0.302 (0.341)	-0.306 (0.333)	-0.784 (0.003)	1.000
Group 2: Girls with %BF ≥ 30 and < 40 (n = 43)						
BW (kg)	0.479 (0.001)	0.928 (< 0.001)	0.947 (< 0.001)	0.947 (< 0.001)	-0.062 (0.694)	-0.329 (0.031)
Height (m)	0.095 (0.543)	0.511 (< 0.001)	0.711 (< 0.001)	0.711 (< 0.001)	-0.021 (0.892)	-0.074 (0.636)
BMI (kg/m^2^)	0.569 (< 0.001)	0.857 (< 0.001)	0.737 (< 0.001)	0.737 (< 0.001)	-0.050 (0.751)	-0.373 (0.014)
Leptin (ng/mL)	0.113 (0.470)	-0.010 (0.948)	-0.104 (0.507)	-0.103 (0.511)	1.000	-0.248 (0.108)
25(OH)D (ng/mL)	-0.059 (0.707)	-0.247 (0.111)	-0.353 (0.020)	-0.353 (0.020)	-0.248 (0.108)	1.000
Group 3: Girls with %BF ≥ 40 (n = 43)						
BW (kg)	0.541 (< 0.001)	0.920 (< 0.001)	0.795 (< 0.001)	0.794 (< 0.001)	0.294 (0.055)	0.295 (0.055)
Height (m)	0.032 (0.838)	0.489 (0.001)	0.826 (< 0.001)	0.825 (< 0.001)	0.265 (0.086)	0.255 (0.098)
BMI (kg/m^2^)	0.721 (< 0.001)	0.900 (< 0.001)	0.477 (0.001)	0.476 (0.001)	0.196 (0.208)	0.204 (0.189)
Leptin (ng/mL)	0.034 (0.831)	0.239 (0.123)	0.281 (0.067)	0.281 (0.068)	1.000	0.247 (0.110)
25(OH)D (ng/mL)	0.096 (0.541)	0.236 (0.128)	0.287 (0.062)	0.287 (0.062)	0.247 (0.110)	1.000

^1^BW, body weight; BMI, body mass index; %BF, body fat percentage; BFM, body fat mass; FFM, fat free mass; MM, muscle mass; 25(OH)D, 25-hydroxyvitamin D.

## Discussion

This study included participants aged 13–14 years, which is the period of age when growth spurt occurs (24). The BMIs for total boys and girls were more than the 97^th^ percentile for age (17), which classified them as obese. Obesity is defined by the WHO as a condition of abnormal or excessive fat accumulation in adipose tissue. However classifying obesity during childhood or adolescence has the added complication of height still increasing, and body composition continually changing (25). In this study, participants in group 1 were defined as having low excess %BF due to %BF < 50^th^ percentile (20), whereas groups 2 and 3 consisted of participants with moderate and high excess %BF, because %BF was above the 95^th^ percentile of %BF-for-age (18, 19). When referring to another percentile curve (20), the mean %BF of girls in groups 2 and 3 was at the 90^th^ and > 95^th^ percentiles, respectively. In each group, BMI did not discern the differences in body composition based on sex (26), and high BMI did not distinguish excess fat mass from lean mass (27). In addition, most variables of body composition varied with the increase in %BF in both sexes, while height, FFM, and MM of boys remained unchanged, which corresponded to growth development during puberty in boys (28). Contrarily, decreases in FFM and MM were observed in girls with the highest excess %BF. However, these characteristics may be related to leptin and 25(OH)D levels which are discussed later.

Furthermore, this study did not find a decrease in 25(OH)D levels due to an increase in %BF as reported in previous studies (13, 29), and 25(OH)D levels appeared to be slightly less than 20 ng/mL in girls with %BF < 40, whereas participants with high excess %BF (≥ 40) had higher serum 25(OH)D levels than the other groups. This finding may be consistent with a previous study reporting that 25(OH)D levels are associated with BMI, sex, puberty, and age (30). The highest 25(OH)D levels were observed in groups with %BF ≥ 40, which might be due to unchanged leptin levels according to the increased %BF, and seemed to be the lowest leptin levels compared to other groups. These results were in contrast to previous findings that serum leptin was increased and that serum vitamin D might be decreased in obese individuals (3, 4, 6, 31). Thus, sex and the amount of %BF are likely the important factors in determining changes in serum leptin and 25(OH)D levels in adolescents.

In this study, boys and girls showed differences in the correlations between leptin, and 25(OH)D levels, likely due to differences in body composition and their relationships in each %BF group. The negative relationship between serum leptin and 25(OH)D was clearly demonstrated in boys with %BF < 40, which was evident in the positive relationships between BMI and body composition and in the inverse relationships with leptin levels. Conversely, the relationship between serum leptin and 25(OH)D was not observed in boys when BMI and excess %BF (> 40) increased, which was likely explained by the change in the lean proportion and the negative relationships between BMI, FFM, and MM. Thus, in boys, lean mass (FFM and MM) and excess BF were likely key variables that indicated a correlation between leptin and 25(OH)D. Although, there are a few studies reporting the relationship between leptin and 25(OH)D in adolescents with obesity, previous studies in adults revealed that measures of adiposity largely explained the negative association of serum 25(OH)D with leptin, and that MM, %BF, and serum 25(OH)D had an impact on serum leptin (5, 32). Furthermore, %BF explained all sex differences in leptin concentrations, and lean body mass was inversely related to leptin concentrations (32).

In girls, whether the inverse relationship between leptin and 25(OH)D was modulated by excess %BF because it appeared in a group with %BF < 30 remained unclear, whereas strong correlations between BMI and body composition were not observed. However, this relationship might be a result of the highest leptin levels and the lowest 25(OH)D levels in girls in this group when compared to other groups, and explained by the excess %BF, thus leading to decreased vitamin D and increased leptin levels (12). In contrast to boys, 25(OH)D levels were inversely related to lean mass (FFM and MM), BW, and BMI in girls with moderately excess %BF. Although, strong correlations between variables of BMI and body composition appeared clearly in girls with high excess %BF, they did not seem to contribute to the relationship between leptin and 25(OH)D. This might be because the 25(OH)D levels in girls did not decrease with increasing %BF, as found in previous studies (12, 13). In addition, the relationship between fat distribution and vitamin D status may be dependent on metabolic factors as well as parathyroid hormone, which is released in response to low 25(OH)D (13). However, this study suggests that BW, BMI, and MM should be considered when interpreting serum 25(OH)D levels as markers of vitamin D status (33).

This study is limited by the small number of participants; thus, it may not be sufficient in powered to be generalizable to Thai early adolescents and to allow comparisons between sexes and groups of excess %BF. Further research is needed to explore the relationship between height, leptin, and 25(OH)D in boys with approximately 30-40%BF, including the inverse relationship between leptin and 25(OH)D in girls.

## Conclusions

Negative correlations between leptin, 25(OH)D, and body composition appeared clearly in boys and girls when using %BF at 30 and 40 to classify their degrees of obesity. The relationship between leptin and 25(OH)D is sex-specific and differ depending on body composition, and the degree of excess body fat may be a determinant.

## Data Availability Statement

The original contributions presented in the study are included in the article/supplementary material. Further inquiries can be directed to the corresponding author.

## Ethics Statement

The studies involving human participants were reviewed and approved by The Human Research Ethics Committee of Walailak University, Thailand (Approval Number: WUEC-19-102-21). Written informed consent to participate in this study was provided by the participants’ legal guardian/next of kin.

## Author Contributions

RK conceived and carried out study design, data collection, data analysis, data interpretation, literature search, and writing of the manuscript. CP carried out ELISA measurement, and involved in writing of the manuscript. All authors contributed to the article and approved the submitted version.

## Funding

The Research Institute for Health Sciences, Walailak University, Thailand under the contract no. WU-IRG-62-018.

## Conflict of Interest

The authors declare that the research was conducted in the absence of any commercial or financial relationships that could be construed as a potential conflict of interest.

## Publisher’s Note

All claims expressed in this article are solely those of the authors and do not necessarily represent those of their affiliated organizations, or those of the publisher, the editors and the reviewers. Any product that may be evaluated in this article, or claim that may be made by its manufacturer, is not guaranteed or endorsed by the publisher.

## Abbreviations

BFM, body fat mass; BMI, body fat index; %BF, body fat percentage; MM, muscle mass; 25(OH)D, 25-hydroxyvitamin D; BW, body weight; FFM, free fat mass; ELISA, Enzyme-Linked Immunosorbent Assay.
